# Exploring the structure and function of *Thermotoga maritima* CorA reveals the mechanism of gating and ion selectivity in Co^2+^/Mg^2+^ transport

**DOI:** 10.1042/BJ20121745

**Published:** 2013-04-12

**Authors:** Nurhuda Nordin, Albert Guskov, Terri Phua, Newsha Sahaf, Yu Xia, Siyan Lu, Hojjat Eshaghi, Said Eshaghi

**Affiliations:** Division of Structural Biology and Biochemistry, School of Biological Sciences, Nanyang Technological University, Singapore 637551, Republic of Singapore

**Keywords:** channel, Co^2+^ transport, gating mechanism, membrane protein, metal ion homoeostasis, Mg^2+^ transport, DDM, dodecyl maltoside, GMN, Gly-Met-Asn, LB, Luria–Bertani, MjCorA, *Methanocaldococcus jannaschii* CorA, Ni-NTA, Ni^2+^-nitrilotriacetate, StCorA, *Salmonella typhimurium* CorA, TM1, first transmembrane helix, TmCorA, *Thermotoga maritima* CorA

## Abstract

The CorA family of divalent cation transporters utilizes Mg^2+^ and Co^2+^ as primary substrates. The molecular mechanism of its function, including ion selectivity and gating, has not been fully characterized. Recently we reported a new structure of a CorA homologue from *Methanocaldococcus jannaschii*, which provided novel structural details that offered the conception of a unique gating mechanism involving conversion of an open hydrophilic gate into a closed hydrophobic one. In the present study we report functional evidence for this novel gating mechanism in the *Thermotoga maritima* CorA together with an improved crystal structure of this CorA to 2.7 Å (1 Å=0.1 nm) resolution. The latter reveals the organization of the selectivity filter to be similar to that of *M. jannaschii* CorA and also the previously unknown organization of the second signature motif of the CorA family. The proposed gating is achieved by a helical rotation upon the binding of a metal ion substrate to the regulatory binding sites. Additionally, our data suggest that the preference of this CorA for Co^2+^ over Mg^2+^ is controlled by the presence of threonine side chains in the channel. Finally, the roles of the intracellular metal-binding sites have been assigned to increased thermostability and regulation of the gating. These mechanisms most likely apply to the entire CorA family as they are regulated by the highly conserved amino acids.

## INTRODUCTION

Mg^2+^ and Co^2+^ are essential ions for all organisms. Mg^2+^ is the most abundant divalent cation among all living organisms and is involved in a myriad of biological activities. Co^2+^ is an essential trace element and, as the cofactor for cobalamin, it is involved in several cellular metabolic pathways, especially the synthesis of DNA and fatty acids. In addition, Co^2+^ is the essential cofactor for many enzymes in several micro-organisms. The intracellular concentrations of these ions must be kept under tight control, as imbalances may lead to major consequences that could ultimately result in cell death. The CorA family of divalent cation transporters is ubiquitous among prokaryotes, with functional homologues in eukaryotes [1–3]. CorA is known to maintain Mg^2+^ homoeostasis in most organisms [1–3] and is suggested to control the concentration of intracellular Co^2+^ in thermophilic Co^2+^-resistant organisms [4]. CorA has also been shown to play an important role in regulating the virulence of pathogens [5–8]. Despite the central role of CorA in the life of various organisms, the details concerning its selection and transport of substrates, and the regulation of the latter, have remained unknown.

The crystal structure of TmCorA (*Thermotoga maritima* CorA) was the first divalent cation transporter structure that became available, presenting a funnel-shaped structure and an apparently closed hydrophobic channel [9–11]. Later, the crystal structure of another Mg^2+^ transporter from the MgtE family was reported [12], which also presented a closed conformation. Nevertheless, none of these structures could reveal how these transporters select and transport their substrates through the lipid bilayer. Moreover, all crystal structures of CorA were lacking the most conserved region of the protein, namely the extracellular loop including the GMN (Gly-Met-Asn) motif, which is considered the signature motif of the CorA family. Nevertheless, the crystal structures of both proteins (CorA and MgtE) share the common feature of intracellular metal-binding sites that were postulated to be involved in the channel gating [13,14]. However, the molecular mechanisms of how CorA performs the gating through the metal-binding sites were still not clear. Recently we have reported the crystal structure of another CorA homologue from the archaea *Methanocaldococcus jannaschii* (MjCorA) (PDB code 4EV6) [15]. This structure provided highly valuable insights into the mechanisms of Mg^2+^ uptake and transport by revealing the structure of the extracellular loop, including the GMN motif. Despite the presence of Mg^2+^ in the channel, the structure of MjCorA also revealed a closed conformation, which was interestingly accompanied by a cluster of Mg^2+^ ions bound to the intracellular binding grooves, but not to the distinct metal-binding sites as found in TmCorA. The Mg^2+^ in the channel appeared to be in a partially hydrated state co-ordinated by polar residues. On the basis of these observations, we proposed a new gating mechanism that involved a helical turn to convert an open hydrophilic pore into a closed hydrophobic one upon metal binding to the intracellular domain. This mechanism was in contradiction to the earlier mechanism proposed by Chakrabarti et al. [13], where a classical hydrophobic gating mechanism was suggested. However, in a recent report the same group has suggested a different mechanism that favours a more complex three-way movement mechanism rather than hydrophobic gating [16]. In the present study, we have further investigated and confirmed our helical turn model through extensive mutagenesis and functional studies on TmCorA. We have also obtained new and more complete structural information concerning the organization of the conserved loop of TmCorA, which shows the same architecture as that of MjCorA as expected and also agrees with our previous postulation that the metal ion is taken up in a partially hydrated form. Furthermore, we have explored in greater detail the role of the metal-binding sites in TmCorA, which reveals different roles for each metal-binding site. Finally, we have explored the ion selectivity of TmCorA to better understand how the selection of either Mg^2+^ or Co^2+^ can be performed. Altogether, the present study, consistent with our previous study, provides new insights into the structure and function of TmCorA, which are expandable to the entire family as these functions are controlled by the most conserved regions of the protein.

## MATERIALS AND METHODS

### Site-directed mutagenesis

The *T. maritima corA* gene was cloned into a pBAD vector as described previously [9]. Site-directed mutagenesis was performed using the QuikChange® kit (Agilent Technologies) as described by the manufacturer. All mutations were validated by DNA sequencing.

### Protein expression and purification

Plasmids carrying the mutant *corA* gene were transformed into *Escherichia coli.* The mutants and the wild-type were overexpressed in *E. coli* and purified as described previously [9], with the exception that 1% DDM (dodecyl maltoside) (Anatrace) was used for solubilization and, subsequently, the detergent concentration was reduced to 0.05% during the purification steps. The His_6_ tag was removed by an off-column cleavage after adding 120 μM TEV (tobacco etch virus) protease (in-house preparation) to the eluate from the Ni-NTA (Ni^2+^-nitrilotriacetate) agarose column (Invitrogen). The solution was incubated at room temperature (20°C) overnight and subsequently run on an Ni-NTA agarose column equilibrated with GF buffer {20 mM Tris/HCl, pH 8.0, 150 mM NaCl, 0.5 mM TCEP [tris-(2-carboxyethyl)phosphine] and 0.05% DDM} to remove the cleaved His_6_ tag from the solution. The eluate was then incubated with 5 mM EDTA to remove any residual divalent metals and desalted with a PD10 column equilibrated with GF buffer. The desalted eluate underwent a final purification step with Superdex 200 16/60 (GE Healthcare) equilibrated with GF buffer.

### SDS/PAGE and Western blot analysis of the whole cells

Whole bacterial cells were adjusted to a *D*_600_ of 1.0 and then subjected to SDS/PAGE using 12% Bis-Tris gels (Invitrogen) according to the manufacturer's recommendations. Protein bands were then transferred from the gel on to nitrocellulose membranes using the I-Blot system (Invitrogen), according to the manufacturer's recommendations. The membranes were then incubated in TBST (150 mM NaCl, 50 mM Tris/HCl, pH 7.5, and 0.05% Tween 20), blocked with 5% BSA (Sigma–Aldrich) and probed with an horseradish peroxidase-conjugated His_6_ probe. West-Pico (Pierce) was used to visualize the bands according to the manufacturer's recommendations.

### Co^2+^ toxicity assay

The assay was performed as described previously [4], with some modifications. The *E. coli* MG1655 with a knockout *corA* (National Institute of Genetics, Mishima, Shizuoka, Japan) was transformed with the mutated *corA* genes. The knock-out *corA* strains transformed with either the empty pBAD vector or *T. maritima corA* were used as negative and positive controls respectively. Cultures were grown overnight at 37°C in LB (Luria–Bertani) medium (Formedium) supplemented with 100 μg/ml each of ampicillin and kanamycin. The overnight culture was diluted 12-fold into 10 ml of fresh LB supplemented with 100 μg/ml each of ampicillin and kanamycin. Cells were left to grow at 37°C for 0.5–2 h depending on the proteins’ particular expression requirements. Protein expression was then induced with 0.02% l-arabinose (Sigma–Aldrich) for 2 h. Subsequently, the cells were harvested at 3000 ***g*** for 10 min at 25°C and washed twice by resuspension in 10 ml of N-buffer [7.5 mM (NH_4_)_2_SO_4_, 5 mM KCl, 1 mM KH_2_PO_4_, 0.5 mM K_2_SO_4_ and 0.1 M Tris/HCl, pH 7.4]. The cells were then normalized to a *D*_600_ of 0.2 by dilution with N-buffer containing various concentrations of Co^2+^. The mixture was then incubated for 10 min at 37°C. Three equivalents of LB containing the same concentrations of Co^2+^ were added into each mixture, and the solution was incubated at 37°C for an additional 3 h. The final *D*_600_ value was recorded and analysed in comparison with the starting *D*_600._ The Mg^2+^ competition assays were conducted similarly by adding Mg^2+^ at various concentrations. All the data and statistics were analysed by Prism 6 (GraphPad).

### Thermostability of CorA mutants in various concentrations of Co^2+^

Purified protein samples suspended in GF buffer were diluted to a concentration of 0.5 mg/ml. The solution was aliquoted into 30 μl volumes, with 1 μl of a stock solution of different Co^2+^ concentrations added to the aliquot. Samples were incubated for 20 min at room temperature and then further incubated for 10 min at every increment of 10°C up to 85°C in a thermocycler (Agilent Technologies). The samples were transferred to a 96-well filter plate (0.65 μm) (Millipore) to remove precipitated proteins. The yield of the filtered protein was analysed by SDS/PAGE stained with Coomassie Brilliant Blue (Merck). Each experiment was carried out at least three times to obtain the S.D.

### Structure determination

TmCorA was crystallized essentially as described previously [9]. Data were collected from three crystals at NSRRC BL13C1 (Taiwan) and the final merged dataset was obtained with XDS [17] at a resolution of 2.7 Å (1 Å=0.1 nm). The structure was solved by the molecular replacement method with PHASER [18] using the previously solved TmCorA structure (PDB code 2IUB) [9] as a search model. The obtained model underwent several rounds of refinement with Phenix software [19] interspersed with manual building in COOT [20]. All structure-related Figures were prepared using PyMOL (http://www.pymol.org). The final structure and structural factors were deposited to the PDB under the accession code 4I0U.

## RESULTS

### The structure of the extracellular ion entrance in TmCorA

**Figure 1:**
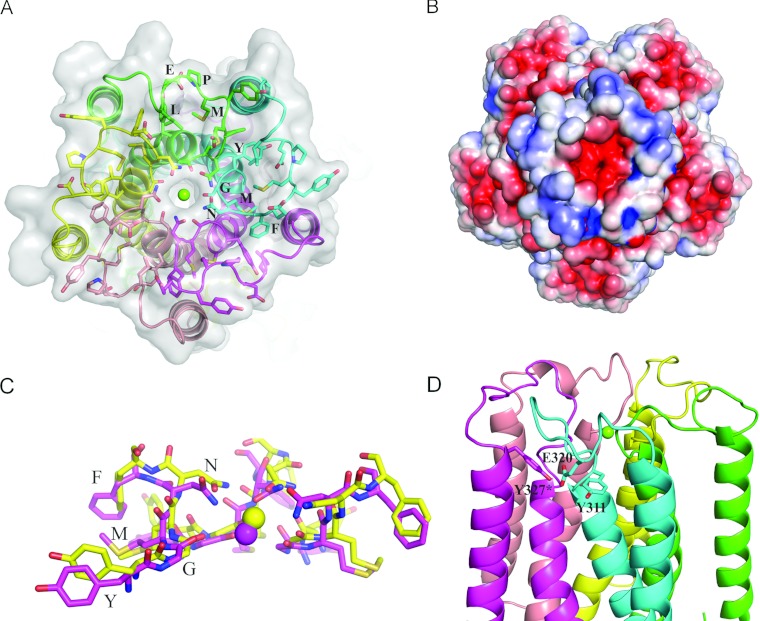
The spatial organization of the extracellular loop in the improved structure of TmCorA (**A**) The periplasmic loop of TmCorA is shown as sticks with the signature motifs YGMNF and MPEL indicated. Protein chains are colour-coded. The magnesium ion is drawn as a green sphere. (**B**) Electrostatic potential surface (±5 kT/e). Note the high negative charge at the selectivity filter. (**C**) Superimposition of YGMNF motifs from MjCorA (yellow) and TmCorA (magenta). RMSD (root mean square deviation) is ~0.6 Å. Three out of five monomers are shown. (**D**) The position of the charged Glu^320^ residue between two conserved tyrosine residues. *From adjacent monomer.

The previously resolved crystal structures of TmCorA [9–11] were all obtained in the closed conformation and show a channel mainly composed of hydrophobic side chains with tight interactions. There are no obvious indications as to how the channel would operate during ion transport, because the structure of the loop was not resolved. In a recent study, we reported the structure of the extracellular entry of MjCorA composed of the GMN motif, where partially hydrated Mg^2+^ was identified as being co-ordinated by the carbonyl groups of glycine and the hydroxy groups of asparagine of the GMN motif [15]. Following a procedure described previously [9], we were able to obtain crystals of TmCorA diffracting up to 2.7 Å (data processing and refinement statistics are given in Table 1). As the data were collected from multiple crystals, we expect some heterogeneity within the data, which in turn has affected the electron density in the extraplasmic loop region. However, for one of the two pentamers in the asymmetric unit, the density was of sufficient quality to assign most of the side chains. The structure revealed an overall arrangement of the periplasmic loop similar to that observed in the MjCorA crystal structure; a concavity stabilized by hydrophobic interactions and relatively high electronegativity towards the channel entry (Figures 1A and 1B, and Supplementary Figure S1 at http://www.biochemj.org/bj/451/bj4510365add.htm). The universally conserved GMN motif is arranged in an almost exact way as that of MjCorA, creating a polar entry made of the Asn^314^ side chain with the underlying ring of the carbonyl group from Gly^312^ (Figure 1C). A strong electron density was found within the pore entry, which was assigned as Mg^2+^ as the crystals were grown in the presence of 100 mM MgCl_2_. It seems that this Mg^2+^ has already passed the Asn^314^ ring and is instead co-ordinated by the carbonyl group of Gly^312^ in a partially hydrated state (distance of ~4 Å), similar to what was seen in MjCorA [15] and in another recent study on TmCorA [16]. The hydrophobic interior part of the loop is further strengthened by Met^311^ as well as Tyr^309^ and Phe^315^, the other two highly conserved residues (the YGMNF motif). Hence the conservation of the structure of the extracellular entry of CorA is now confirmed by comparing the new improved structure of TmCorA with the recently reported structure of MjCorA.

**Table 1:** Data collection and refinement statistics The highest resolution shell is shown in parentheses. PEG, poly(ethylene glycol); RMSD, root mean square deviation.

Parameter	Value
Data collection	
Space group	*P*2_1_
Cell dimensions	
*a*, *b*, *c* (Å)	116.25, 151.50, 143.36
α, β, γ (°)	90.0, 98.9, 90.0
Numbers of reflections measured	608 068
Number of unique reflections	121 642
Resolution (Å)	40–2.7 (2.8–2.7)
*R*_merge_	0.16 (0.67)
*I*/σ*I*	6.8 (1.73)
Completeness (%)	90.5 (87.7)
Redundancy	4.99 (3.8)
Refinement	
Resolution (Å)	38.3–2.7
Number of reflections (test set)	121 600 (6079)
*R*_work_/*R*_free_	22.80/28.91
Number of atoms	
Protein	28 559
PEG/detergent/ion/hydrated ion	155/102/17/101
Water	161
*B*-factors	
Protein	77.34
PEG/detergent/ion/hydrated ion	88.38/136.29/65.81/46.37
Water	54.58
RMSD	
Bond lengths (Å)	0.011
Bond angles (°)	1.610
Ramachandran plot statistics (%)	
Favoured regions	93.16
Allowed regions	6.05
Disallowed regions	0.79

The major differences between the loops of TmCorA and MjCorA are after the YGMNF motif. In MjCorA, this stretch is composed of SYLPLA (which is also less conserved within the family), whereas TmCorA contains the conserved region EYMPEL (where MPEL is the second signature motif of the CorA family). The latter is thus more charged compared with the more hydrophobic motif in MjCorA. The structure of the TmCorA loop reveals that the negatively charged Glu^316^ faces towards the concavity, and most probably facilitates the initial trawling of Mg^2+^ ions, as predicted previously [15]. Biochemical studies as well as molecular dynamics simulations have suggested Glu^316^ to be the main binding and selection site for a fully hydrated Mg^2+^ [21,22]. However, on the basis of the crystal structures of MjCorA and TmCorA loops, this residue plays a rather auxiliary role to trap the ion substrate (which is in its fully hydrated form), but the actual selection is performed by the polar asparagine ring. More puzzling is the positioning of the second charged residue (namely Glu^320^) of the conserved MPEL motif. It is facing away from the concavity and is trapped in a hydrophobic patch surrounded by highly conserved tyrosine and proline residues (Tyr^311^, Pro^319^ and Tyr^327^*, where * denotes the residue from the adjacent monomer) (Figure 1D) as well as lipid molecules. This arrangement does not seem stable, at least not to the same degree as in MjCorA involving the LPLA motif.

### The TmCorA substrate passes through a polar channel gated by helical rotation

**Figure 2:**
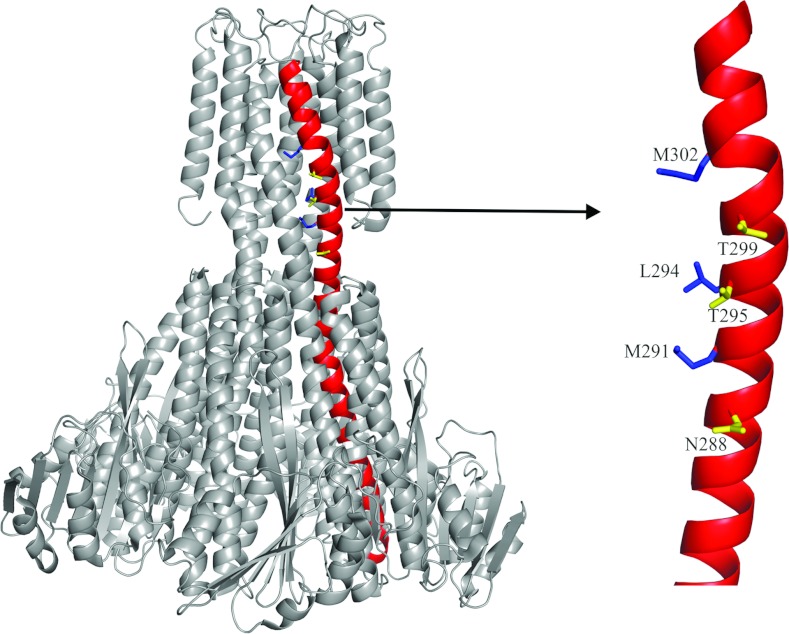
The arrangement of hydrophobic and polar residues on helix 7 The structure of the pentameric TmCorA is presented in grey with helix 7 highlighted in red. The zooming of the transmembrane region of helix 7 shows the vertical alignment of both the hydrophobic residues (blue) exposed to the pore and the polar residues (yellow) facing away from the pore, in the closed conformation.

A vertical alignment of three conserved polar residues (Asn^288^, Thr^295^ and Thr^299^) was identified on the first transmembrane helix (TM1), close to the hydrophobic constriction site (Figure 2). These residues face away from the pore; however, a counter-clockwise rotation of helix 7, and thus TM1, would place them inside the pore. Such rotation and positioning of the residues would create a polar environment suitable for partially hydrated Co^2+^ to pass through. On the basis of this hypothesis, a replacement of these polar residues with hydrophobic ones should abort transport.

**Figure 3:**
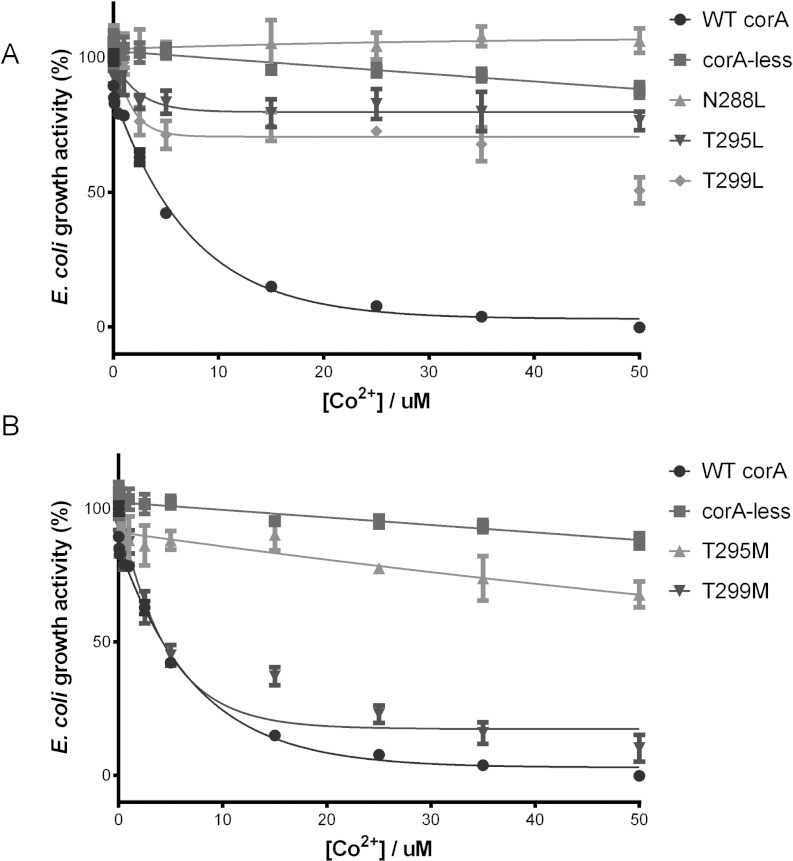
The involvement of the polar residues in Co^2+^ transport The growth activity of TmCorA with the Asn^288^, Thr^295^ and Thr^299^ mutated to either (**A**) leucine or (**B**) methionine was monitored in the presence of various Co^2+^ concentrations. A reduction in growth activity upon Co^2+^ concentration increase is indicative of the Co^2+^ transport activity of the TmCorA variant. The wild-type TmCorA (WT corA) and the empty CorA-less pBAD vector (corA-less) were used as positive and negative controls respectively. The results are the means±S.D. of at least three independent experiments.

To validate our hypothesis, we performed site-directed mutagenesis on Asn^288^, Thr^295^ and Thr^299^ to create either leucine or methionine residue mutations, since the closed conformation of the channel is composed of these hydrophobic residues. The N288L, T295L and T299L mutants showed quite similar protein expression as the wild-type TmCorA during the large-scale purification of the membrane fractions. Additionally, the channel intactness was verified using size-exclusion chromatography (Supplementary Figure S2 at http://www.biochemj.org/bj/451/bj4510365add.htm). To study the Co^2+^ transport activity, we used our previously reported Co^2+^ transport assay, where a *corA*-deficient *E. coli* strain that is resistant to Co^2+^ becomes highly Co^2+^-sensitive upon expressing recombinant TmCorA [4]. TmCorA, when carrying the N288L single mutation alone or in combination with the T295L and T299L mutations, failed to demonstrate Co^2+^ sensitivity, indicating that the leucine residue mutation(s) had completely blocked Co^2+^ transport (Figure 3A). The single mutations of T295L and T299L caused the inhibition of Co^2+^ transport to approximately 80% and 50% of original levels respectively (Figure 3A). The double and triple mutations, however, completely inhibited Co^2+^ transport in the same way as the N288L mutation (Supplementary Figure S3 at http://www.biochemj.org/bj/451/bj4510365add.htm). These data suggest that these three residues are essential to the function of the transporter. It is likely that they face the channel and create a polar environment suitable for transport.

As the leucine-mediated blockage of the channel decreases towards the Thr^299^ site, it could indicate that the ion passage through the pore is narrowest at the cytoplasm/membrane interface (at Asn^288^) and becomes wider towards Thr^299^. This interface is in turn comparable with the size and shape of the pore in its closed conformation. To further explore this idea, we performed the Co^2+^ transport assay on TmCorA bearing either the T295M or the T299M mutations. The inhibitory effect of the T295M mutation on the TmCorA Co^2+^ transport was almost identical with that of the T295L mutation (Figure 3B). However, the T299M mutation did not cause significant inhibition and the mutated TmCorA showed a transport activity similar to the wild-type TmCorA (Figure 3B). Methionine, although being longer than leucine, is less rigid and slightly polar due to its sulfur group. Therefore the inability of the T299M mutation to match the mutation's ability to close the pore ultimately strengthens the perception that the open channel diameter of the Thr^299^ region is wider than that of the Thr^295^–Asn^288^ stretch. The partial loss of function of T295M and especially T299M further supports that the leucine mutations have blocked the channel. It is not surprising that methionine mutations are able to facilitate Co^2+^ transport at least to some degree, as the polarity of the methionine sulfur group has been shown to allow the binding of metal ions (such as silver and copper) and aids their transport [23].

**Figure 4:**
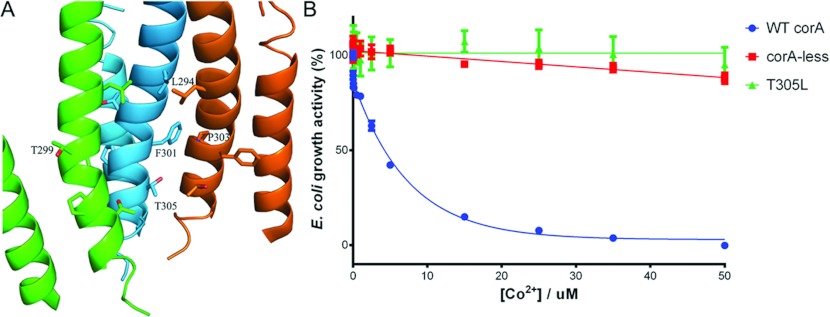
Involvement of Thr^305^ in Co^2+^ transport (**A**) The structural arrangement of Thr^305^ inside the channel and its position relative to Leu^294^ and Thr^299^. The Phe^301^–Phe^303^ kink-forming site is positioned between Leu^294^/Thr^299^ and Thr^305^. For clarity, only three monomers are included. (**B**) The growth activity of the T305L mutant was monitored in the presence of various Co^2+^ concentrations. A reduction in growth activity upon Co^2+^ concentration increase is indicative of the Co^2+^ transport activity of the TmCorA variant. The wild-type TmCorA (WT corA) and the empty CorA-less pBAD vector (corA-less) were used as positive and negative controls respectively. The results are the means±S.D. of at least three independent experiments.

According to the crystal structure, Thr^305^ is the lone polar residue inside the channel. A corresponding threonine residue in the MjCorA structure, Thr^264^, was observed to mediate Mg^2+^ binding in the closed conformation, thus it is possible that Thr^305^ of TmCorA performs the similar role. To verify whether its hydroxy group is positioned in the channel in the open conformation of TmCorA, we mutated this threonine residue to a leucine. If TmCorA with the T305L mutation displays resistance to Co^2+^, it would indicate that Thr^305^ faces the channel during ion transport and thus is indeed involved in the ion co-ordination. In fact, the Co^2+^ transport assay revealed that the T305L mutation inhibited the transport ability of TmCorA (Figure 4B), which is a clear indication that the position of Thr^305^ remains unchanged during the operation of the gate. Thus the helical rotation must stop at the Thr^299^/Met^302^ site.

### Threonine residues in the channel determine the Co^2+^ selectivity of TmCorA

**Figure 5:**
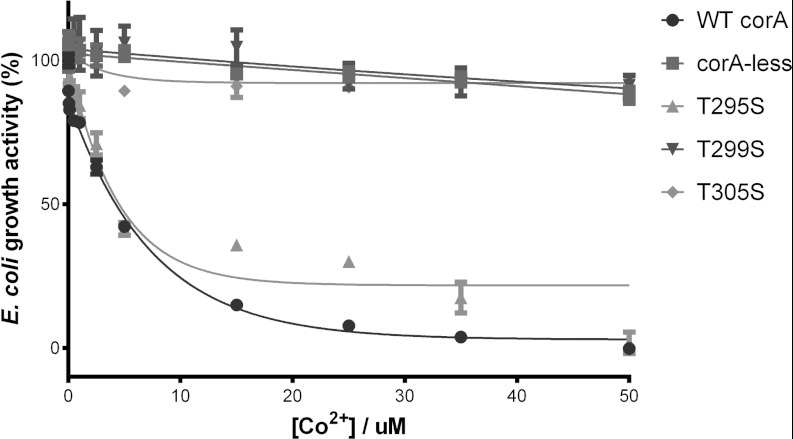
Exploring the Co^2+^ transport ability of threonine residues in the channel The growth activity of TmCorA with the T295S, T299S or T305S mutant was monitored in the presence of various Co^2+^ concentrations. A reduction in growth activity upon Co^2+^ concentration increase is indicative of the Co^2+^ transport activity of the TmCorA variant. The wild-type TmCorA (WT corA) and the empty CorA-less pBAD vector (corA-less) were used as positive and negative controls respectively. The results are the means±S.D. of at least three independent experiments.

**Figure 6:**
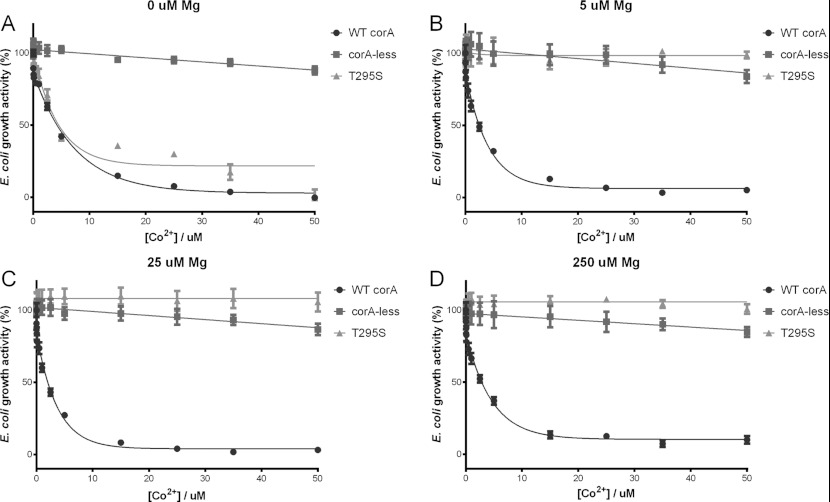
Assaying the sensitivity of the T295S mutant towards Mg^2+^ The growth activity of the T295S mutant was monitored in the presence of increasing Co^2+^ concentrations, as well as a fixed Mg^2+^ concentration of (**A**) 0 μM, (**B**) 5 μM, (**C**) 25 μM and (**D**) 250 μM. A reduction in growth activity upon Co^2+^ concentration increase is indicative of the Co^2+^ transport activity of the TmCorA variant. The wild-type TmCorA (WT corA) and the empty CorA-less pBAD vector (corA-less) were used as positive and negative controls respectively. The results are the means±S.D. of at least three independent experiments.

Asn^288^ of TmCorA is the most conserved residue in TM1 in the CorA family, including both the A and B subgroups [2]. Thr^299^ and Thr^295^ are also highly conserved, but only in subgroup A. Their counterparts in subgroup B, however, are serine residues. Subgroup B includes channels that have been shown to be Mg^2+^-selective, such as StCorA (*Salmonella typhimurium* CorA), *E. coli* CorA and *Haemophilus influenzae* CorA, whereas the TmCorA of subgroup A is a Co^2+^-selective transporter. The presence of threonine in subgroup A and serine in subgroup B appears to be the only main difference in the polar side chains that are active in ion transport. We hypothesized that the differences in substrate specificity between the two subgroups could be due to the presence of threonine or serine residues. To test this hypothesis, we performed site-directed mutagenesis to create the T305S, T299S and T295S mutations in TmCorA. These mutants were then subjected to the Co^2+^-transport assay. As shown in Figure 5, the Co^2+^ transport activity of TmCorA carrying the T295S mutation did not dramatically affect Co^2+^ sensitivity. However, both the T305S and T299S mutations completely impaired Co^2+^ transport. This finding clearly indicates the importance of threonine residues in Co^2+^ selection. Thr^305^ and Thr^299^ are situated closer to the periplasmic entrance, hence they are apparently more effective in ion selection. Thr^295^ is situated much farther from the channel entrance, which may prevent it from affecting Co^2+^ selection. Nevertheless, to further explore whether T295S is less Co^2+^-selective, and also whether the presence of serine in TM1 can increase the Mg^2+^-selectivity of CorA, we included Mg^2+^ as a competitor to Co^2+^ at various concentrations while assaying the Co^2+^ transport activity of the T295S mutant. Already in the presence of 5 μM Mg^2+^, the T295S channel was unable to select for Co^2+^, whereas wild-type TmCorA was highly sensitive to low concentrations of Co^2+^, even in the presence of 250 μM Mg^2+^ (Figure 6), in the same manner as reported previously [4]. These data strongly indicate that the threonine residues in the TmCorA channel are not only involved in ion co-ordination, but also control Co^2+^ selection. The data also suggest that Mg^2+^ is better selected by serine residues, explaining the presence of serine in the transmembrane region of Mg^2+^-selective transporters.

### A cytoplasmic metal-binding site aids in keeping TmCorA stable and functional

**Figure 7:**
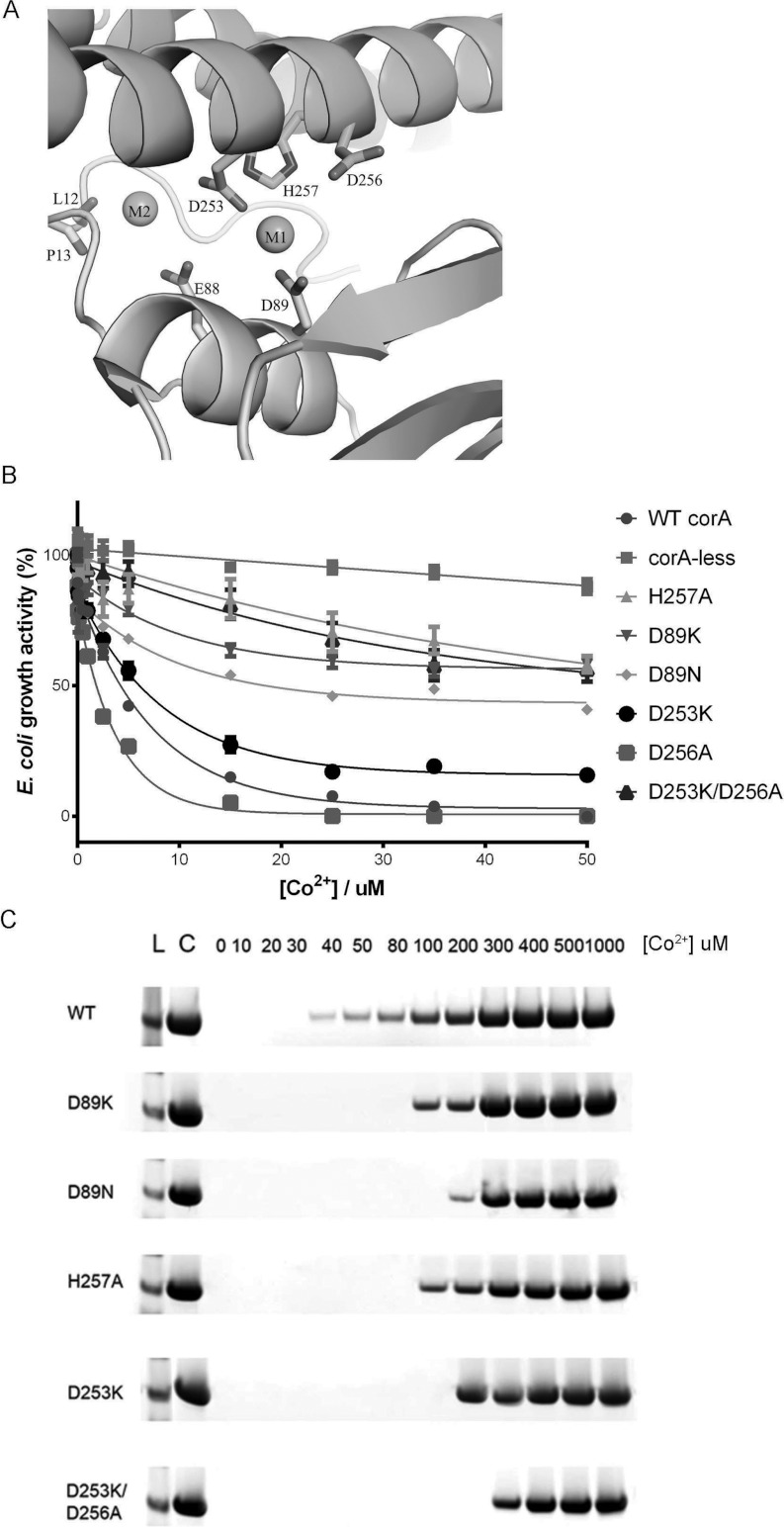
The involvement of the M1-site in the stability and activity of TmCorA (**A**) The structure of the M1-site with a Co^2+^ and the co-ordinating side chains. (**B**) The growth activity of TmCorA with the M1-site specific mutants was monitored in the presence of various Co^2+^ concentrations. A reduction in growth activity upon Co^2+^ concentration increase (Co^2+^ toxicity) is indicative of the Co^2+^ transport activity of the TmCorA variant. The wild-type TmCorA (WT corA) and the empty CorA-less pBAD vector (corA-less) were used as positive and negative controls respectively. The results are the means±S.D. of at least three independent experiments. (**C**) Thermostability of TmCorA wild-type and the M-site mutants in the presence of various Co^2+^ concentrations. Each sample was heated at 85°C for 10 min and the presence of stable protein was analysed by SDS/PAGE followed by Coomassie Blue staining. Untreated TmCorA was used as a positive control. The protein size was verified using Seeblue® Plus2 Pre-stained Standard (Invitrogen) (L). The concentration of Co^2+^ used is indicated from 0 to 1000 μM.

An anti-clockwise rotation along helix 7 of TmCorA would also cause the disruption of metal co-ordination in the metal-binding sites, M1 and M2, as observed in the closed conformation (Figure 7A). Thus, in the open channel, the metal-binding sites are presumably unoccupied and the polar residues face the interior of the channel, such that the occupancy of the M1 and M2 sites will cause a helical turn and close the channel. The crystal structure (PDB code 2IUB) [9] revealed Co^2+^ binding at the M1 site that is tightly co-ordinated by the carboxyl groups of Asp^89^ and Asp^253^. The distance between Co^2+^ and these carboxyl groups is approximately 2 Å, thus excluding any bridging water molecules. If the binding of Co^2+^ to the M1 and M2 sites is required to close the channel, then a disruption in metal co-ordination would result in a permanently open channel. Hence a Co^2+^ sensitivity that is similar to or worse than that of wild-type TmCorA will be detected. The D89K mutation resulted in the reverse effect, with significantly reduced Co^2+^ sensitivity, indicating impaired Co^2+^ transport (Figure 7B). This mutation may have created a salt bridge with Asp^253^ and thus triggered channel closure, as suggested earlier by Payandeh et al. [24]. To verify this, we analysed the Co^2+^ transport ability of TmCorA carrying the D89N mutation. Similar to the D89K mutation, the D89N mutation did not trigger Co^2+^ sensitivity to the same degree as the wild-type TmCorA (Figure 7B). These results demonstrate the importance of Asp^89^ for the stability of TmCorA. We recently showed that the stability of TmCorA at physiological temperatures was dependent on the presence of its Co^2+^ substrate, without which it stays stable only up to 75°C; the addition of micromolar Co^2+^ ensured TmCorA stability up to 95°C [4]. Thermostability analysis revealed that both the D89K and D89N mutations significantly reduced TmCorA's thermostability (Figure 7C). Taken together, these data suggest that Asp^89^ is essential for both the functionality and stability of TmCorA, in which the latter is also dependent on the presence of Co^2+^. As Asp^89^ is clearly involved in the co-ordination of the Co^2+^ bound to the M1 site, its importance in maintaining stability and functionality must be directly linked with the role of the M1-site in the stability of TmCorA. To test this further, we performed additional site-directed mutagenesis to specifically target the M1-site. His^257^ appears to co-ordinate the Co^2+^ in the M1-site (Figure 7A). Owing to the ability of histidine to ordinarily ligate Co^2+^, in contrast with Mg^2+^, where such a type of co-ordination is common only in chlorophylls [25], His^257^ could define the specificity of the M1-site for Co^2+^. Hence the H257A TmCorA mutant was created and its Co^2+^ transport ability was analysed. The experiment showed that mutation at this position affects the transport ability of TmCorA in a more pronounced manner than the Asp^89^ mutations (Figure 7B). Additionally, the stability of TmCorA was reduced with the H257A mutation (Figure 7C). Finally, we examined the importance of Asp^253^ for the functionality and stability of TmCorA. The D253K mutation did not cause any significant change in the Co^2+^ transport activity of TmCorA (Figure 7B). This residue is shared between both the M1- and M2-binding sites. Additionally, as observed in the crystal structure, another aspartate residue, Asp^256^, is located adjacent to the M1-site, but not to the M2-site. This residue could be involved in Co^2+^ co-ordination at the M1-site, in the absence of Asp^253^. Therefore we created the double mutation D253K/D256A. The Co^2+^ transport assay revealed that this double mutation made TmCorA more resistant to Co^2+^ in a similar manner to the H257A mutation (Figure 7B). It was clear that this effect was only caused by disrupting both Asp^253^ and Asp^256^, as the single mutations failed to significantly affect the transport activity of TmCorA. Collectively, the M1-binding site was identified as the stabilizing metal-binding site, which needs to be intact, and perhaps constantly occupied by Co^2+^, to maintain the functionality of TmCorA.

**Figure 8:**
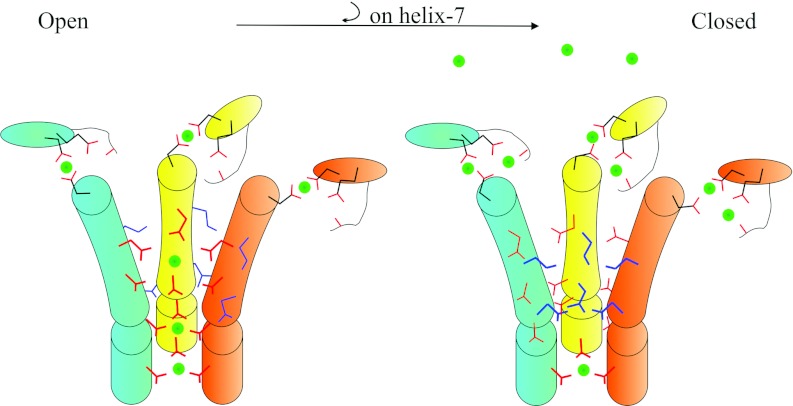
Illustrative model of the mechanisms of Co^2+^ transport and gating by TmCorA The cartoons represent helix 7 (cylinders) and the α-β regions (ovals) from three (out of five) monomers (cyan, yellow and orange). When the channel is open, the hydroxy-group-containing side chains of Thr^305^, Thr^299^, Thr^295^ and Asn^288^ (red lines) face the channel (thicker lines) and co-ordinate the transport of partially hydrated Co^2+^ (green circles). The M1 site is also occupied by Co^2+^ through tight interactions with Asn^89^ (on the α-β region) and Asp^253^ (on helix 7). Co^2+^, once reaching high concentrations within the cytoplasm, occupies the M2 site. This binding pulls Asp^253^ and causes a clockwise rotation of helix 7 along its axis. This rotation will then remove the polar residues from the channel and replace them with the hydrophobic residues (blue lines), which prevents ion movement through the channel.

Altogether, the mechanism of ion transport and gating can be summarized as illustrated in Figure 8: when TmCorA is open, partially hydrated Co^2+^ passes through a polar channel co-ordinated by hydroxy groups of threonine and asparagine; threonine is also instrumental to select for Co^2+^. The metal-binding site M1 is constantly and tightly occupied to maintain the stability and functionality of TmCorA. When the intracellular concentration of Co^2+^ is elevated, Co^2+^ also binds to the M2 site. This binding causes helix 7 to turn clockwise, which sequentially will result in the removal of the polar residues from the channel and replace them with hydrophobic leucine and methionine residues, and thus this will close the gate.

## DISCUSSION

As seen in the crystal structure, the pore is polar near the entrance at residue Thr^305^. Most likely, this polarity begins at the entry with the conserved Asn^314^ from the GMN motif. From Met^302^ towards the cytoplasmic side, the diameter of the pore decreases and the interior residues become completely hydrophobic. A hydrophobic pore is completely closed to ions with radii of 4.5 Å or smaller [26]. Such a pore partially opens if the radius is increased to approximately 5.5 Å and will completely open to ions at a radius of 7 Å or larger, at which point the ions move freely inside the bulk water [26]. Chakrabarti et al. [13] have postulated that the opening of the CorA channel is driven by the widening of the hydrophobic pore. However, it is currently unclear how the increase in channel radii can occur by metal ion binding to the cytoplasmic regulatory domain. More recently the same group suggested another mechanism partially abolishing their previous model [16]. Their new hypothesis is that channel gating occurs as a series of complex movements consisting of radial tilt, lateral movement and z-rotation (along the channel axis). The latter movement most probably corresponds to our proposed helical turn. However, these postulations are based on low-resolution structures (3.8 Å and 3.9 Å) of a construct that lacks the N-terminal domain of the protein (which is likely to be important for the M2-site) and also contains two single mutations on helix 7; and their activity assays showed this construct to be non-functional. Hence, these different models clearly need much more supporting functional and structural data to be validated. In contrast with the previous hypothesis, a polar pore need only be partially open to ions at approximately 3 Å in radius [26]. Therefore a conversion of a narrow hydrophobic pore (<2.5 Å in radius) to a polar pore would result in the opening of the pore to ions. We have shown that this is exactly what happens in TmCorA, as the hydroxy groups of Thr^299^, Thr^295^ and Asn^288^, along with that of Thr^305^, are aligned on helix 7 and face the channel. This is consistent with our recent model based on the crystal structure of MjCorA, where we also identified partially hydrated Mg^2+^ within the channel co-ordinated by polar residues [15]. A partially hydrated metal ion does not bind to hydroxy groups strongly, but rather undergoes a transient interaction. The latter permits a much more efficient on/off rate and thus the rate of transport will be increased. This could be the reason for the high influx rate reported for Mg^2+^ transport in StCorA [21]. Most likely, this is why hydroxy groups are involved in Co^2+^ and Mg^2+^ transport and not the negatively charged carboxyl or carbonyl groups. Accordingly, a clockwise turn along helix 7 will remove Thr^299^, Thr^295^ and Asn^288^ from the pore and replace them with mainly Met^302^, Leu^294^ and Met^291^ to close the pore. Presumably, such a rotation would be more energetically favourable than the sideway movements of helices, or combination of co-ordinated movement in three directions assured by breaking and forming pairs of salt bridges. However, the position of Thr^305^ is not changed and the helical turn appears to be stopped at the Thr^299^/Met^302^ site. This stop appears to be mediated by the hydrophobic interactions between the side chains of Phe^301^ of one monomer and Pro^303^ of an adjacent monomer, which creates a kink in the TM1 (Figure 4A). A similar kink was also observed in the MjCorA structure [15]. The reasons for this kink/stop could be to avoid disrupting the entrance architecture, and/or to shorten the helix turn distance and thereby minimizing energy requirements. Upon the helical turn along helix 7, a number of hydrogen bonds are most likely broken, while new hydrogen bonds are formed. Owing to the absence of the structure of CorA in open conformation, we do not know the exact co-ordination of helix 7 when the channel is open and thus do not know the degree of the rotation. Therefore with the currently available structural data it is very difficult to predict the required energy to initiate the rotation and break hydrogen bonds. However, assuming the gating of the channel is fully reversible, the net energy required for breaking and remaking hydrogen bonds should be equal to zero. Hence the binding of ion to the M2 site should provide sufficient energy to initiate the rotation. Certainly, structural data concerning the open conformation as well as thorough molecular dynamics simulation studies are required to gain reliable understanding concerning the energy requirements for this gating mechanism.

The position of the negatively charged Glu^320^ of the conserved MPEL motif located in the periplasmic loop is puzzling. This motif is highly conserved and preferred among bacteria. The side chain of Glu^320^ is positioned towards the hydrophobic environment. Such a non-favoured location of Glu^320^ might make the loop less rigid and more flexible than the corresponding loop in MjCorA. This might explain why it was tremendously difficult to get the structure of the periplasmic loop in the case of TmCorA, compared with MjCorA where MPEL is substituted with the LPLA motif, which provides the extreme rigidness of the loop. Perhaps such flexibility will in turn allow for better movements, either during the ion uptake, or during the helical rotation and gating.

All members of the CorA family characterized so far have shown the ability to transport both Mg^2+^ and Co^2+^. However, TmCorA from subgroup A has shown high selectivity for Co^2+^ over Mg^2+^, whereas members from subgroup B, such as StCorA, have shown a stronger preference for Mg^2+^. The entrance of all CorAs is made of highly conserved residues and therefore the selection between Mg^2+^ and Co^2+^ is most likely not at the entrance. Our data show that the Co^2+^ selectivity of TmCorA is highly dependent on the presence of threonine residues in the channel. The fact that removal of the methyl group from the threonine residues in the channel turns TmCorA into being more selective for Mg^2+^ is certainly remarkable and intriguing. In line with that, the pore-forming TM1 contains more serine than threonine residues in the Mg^2+^-selective subgroup B. Both Mg^2+^ and Co^2+^ have six water molecules in their first hydration shell [27,28]. However, Mg^2+^ has been consistently reported to contain 12 water molecules in its second hydration shell, whereas for Co^2+^ this number has varied from six to 14 (see [27,28] and the references therein). Although the accuracy of the methods used to quantify the number of water molecules in the second hydration shell can be questionable, there has been a consistency in the numbers reported for Mg^2+^, but not for Co^2+^. Thus it is reasonable to assume that perhaps Co^2+^ has a more flexible second hydration shell as compared with Mg^2+^. The rigidity in the second hydration shell of Mg^2+^ may prevent it from readily interacting with threonine, which contains a bulky methyl group, as compared with that of serine.

The specificity of TmCorA for Co^2+^ is not limited to the threonine residues in the channel. The regulatory cytoplasmic metal-binding site is also Co^2+^-specific, as shown by various competition studies as well as thermostability studies, in which Co^2+^ consistently outcompeted Mg^2+^ [4,9]. The gating mechanism proposed in the present study is regulated by the binding of Co^2+^ to these sites. Hence, during ion transport, these sites would be unoccupied; upon an increase in intracellular Co^2+^, the ions would bind to these sites, for which the co-ordinating residues on helix 7 would need to turn clockwise. Nevertheless, some considerations could render such a sequence of events rather debatable. For example, the metal ion in M1 binds directly to Asp^89^ and Asp^253^, as their carboxyl groups replace the water molecules of the first hydration shell. Such dehydration requires a significant energy input; likewise, the rehydration would also cost additional energy. The latter could lead to the question of whether the M1 site is always occupied and holds a different role other than regulating the gating process. As has been shown, TmCorA requires Co^2+^ to remain stable at physiological temperatures [4]. Disrupting the M1 site directly destabilizes TmCorA, which in turn affects the functionality of the channel. These effects are more pronounced when the amino acids specific to M1 are mutated, such as Asp^89^ and His^257^. The latter is highly conserved in subgroup A of the CorA family, to which the Co^2+^ transporter TmCorA belongs; the residue is missing in subgroup B of the Mg^2+^ transporters, as well as in the archaean CorA from MjCorA, which is also a selective Mg^2+^ transporter. This organism is also hyperthermophilic and prefers optimal surrounding temperatures of up to 94°C [29]. Our thermostability tests on MjCorA revealed that this protein is highly thermostable regardless of the presence of Mg^2+^ [15], contrary to the Co^2+^ requirement for the thermostability of TmCorA. Therefore we believe that the M1 site is indeed always occupied with Co^2+^ and its main role is to maintain the stability and thus the functionality of TmCorA. When the intracellular Co^2+^ concentration is increased, it will bind to the M2 site. The M2 site, in contrast with the M1 site, is occupied by a partially hydrated ion, which binds to the co-ordinating amino acids via transient hydrogen bonds. The occupation of the M2 site will then induce a pulling force, which may be enhanced by the occupancy of the M1 site. The latter reasoning is based on the involvement of Asp^253^ in co-ordinating the ions in both sites. This pulling force is supported by the carbonyls from the backbone of Leu^12^ and Pro^13^ (Figure 7A). These amino acids are part of the extra-long N-terminal domain of TmCorA, which is present in subgroup A, but not in the Mg^2+^ transporters of subgroup B and MjCorA.

The structure of the ion pathway and the gating mechanism of the TmCorA all involve highly conserved amino acids within the CorA family. Therefore the functional mechanisms suggested in the present study most likely represent those of the entire family. In addition, the differences within the CorA subgroups reflect their unique differences to suit their physiological roles.

In conclusion, CorA transports its substrate in a partially hydrated form. A novel gating mechanism, in which substrate binding to the cytoplasmic binding sites converts a narrow open hydrophilic pore into a narrow closed hydrophobic pore, regulates this transport. The presence of threonine or serine residues in the CorA channel determines the preference of the channel for either Co^2+^ or Mg^2+^.

## Online data

Supplementary data
