# Supplementary material for: Exploring the structure and function of Thermotoga maritima CorA reveals the mechanism of gating and ion selectivity in Co2+/Mg2+ transport
Source: Biochem J. 2013 Apr 12;451(Pt 3):365–74. doi: 10.1042/BJ20121745 (PMC3629940; doi:10.1042/BJ20121745)
Supplement: Supplementary data [file bj4510365add.pdf]

Biochem. J. (2013) **451**, 365–374 (Printed in Great Britain) doi:10.1042/BJ20121745

SUPPLEMENTARY ONLINE DATA

Exploring the structure and function of *Thermotoga maritima* CorA reveals the mechanism of gating and ion selectivity in Co^{2+}/Mg^{2+} transport

Nurhuda NORDIN, Albert GUSKOV, Terri PHUA, Newsha SAHAF, Yu XIA, Siyan LU, Hojjat ESHAGHI and Said ESHAGHI[1]

Division of Structural Biology and Biochemistry, School of Biological Sciences, Nanyang Technological University, Singapore 637551, Republic of Singapore

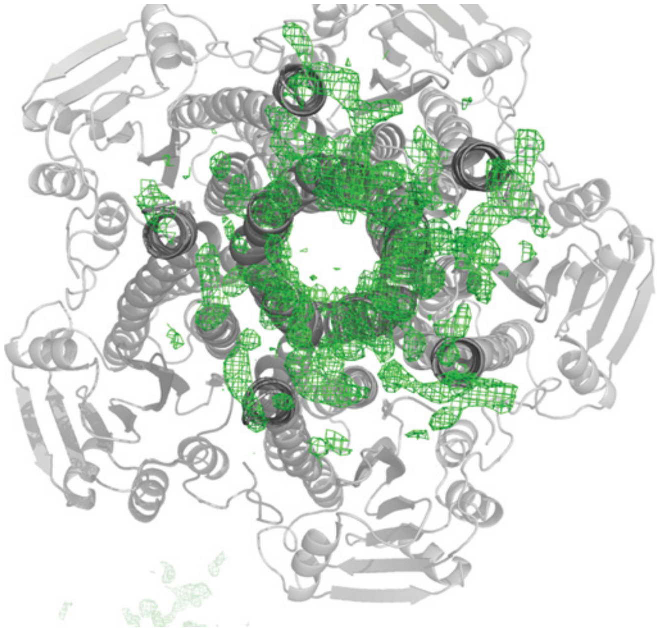

Figure S1 Electron density of the periplasmic loop

Example of electron density ($F_o - F_c$) in the area of periplasmic loop observed just after the molecular replacement and rigid body refinement before modelling of the loop. σ cut-off = 2.5

[1] To whom correspondence should be addressed (email said.eshaghi@ntu.edu.sg).

The structural co-ordinates reported for *Thermotoga maritima* CorA will appear in the PDB under accession code 4I0U.

© 2013 The Author(s)

The author(s) has paid for this article to be freely available under the terms of the Creative Commons Attribution Licence (CC-BY) (http://creativecommons.org/licenses/by/3.0/) which permits unrestricted use, distribution and reproduction in any medium, provided the original work is properly cited.

Biochemical Journal www.biochemj.org

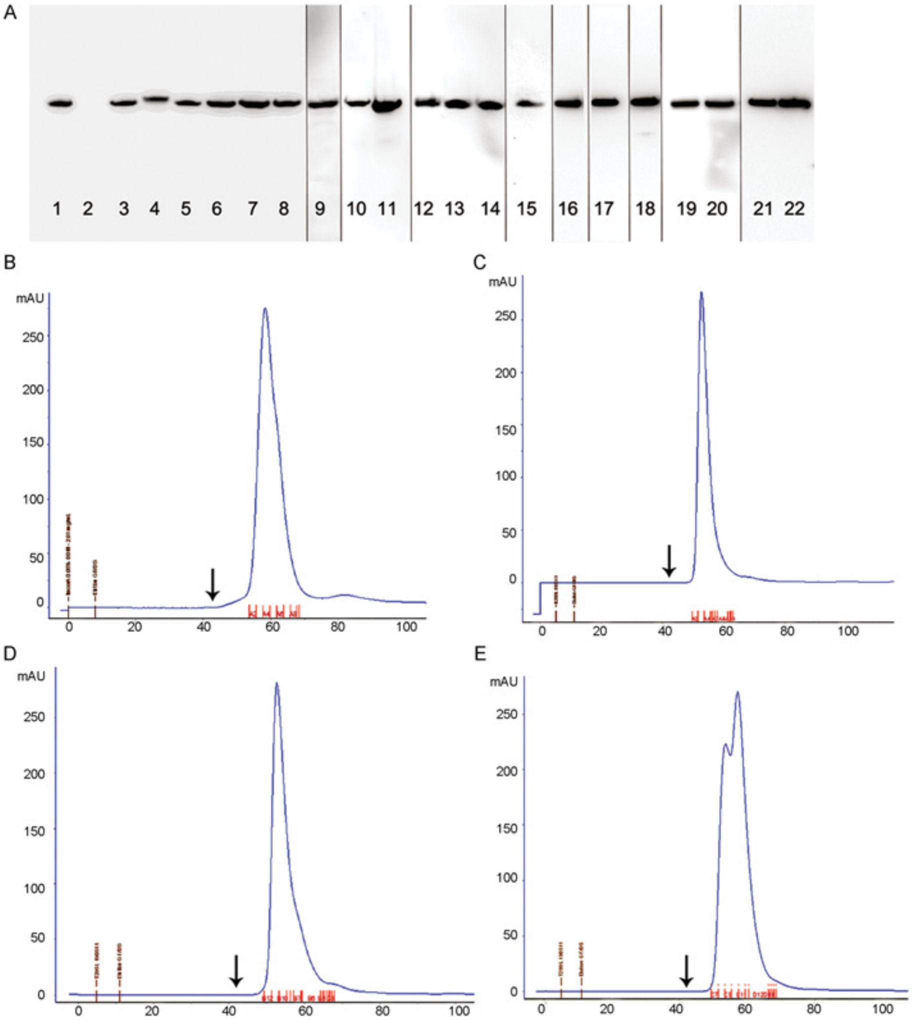

Figure S2 Quality control of the mutations produced

(**A**) Western blot of the whole cell protein expression of the different TmCorA mutants. Lane 1, wild-type TmCorA; lane 2, *corA*-less *E. coli*; lane 3, T295L; lane 4, T299L; lane 5, N288L; lane 6, N288L/T295L; lane 7, N288L/T299L; lane 8, N288L/T295L/T299L; lane 9, T305L; lane 10, T295M; lane 11, T299M; lane 12, T305S; lane 13, T295S; lane 14, T299S; lane 15, L294N; lane 16, D89K; lane 17, D89N; lane 18, H257A; lane 19, D253K; lane 20, D256A; lane 21, D253A/D256A; and lane 22, D253K/D256A. Black lines separate different Western blot membranes from each other. However, the expression level of the wild-type TmCorA was the same in all the experiments. (**B**–**E**) The gel-filtration profiles of (**B**) wild-type TmCorA, (**C**) N288L, (**D**) T295L and (**E**) T299L mutants. The void volume is indicated by an arrow.

© 2013 The Author(s)
The author(s) has paid for this article to be freely available under the terms of the Creative Commons Attribution Licence (CC-BY) (http://creativecommons.org/licenses/by/3.0/) which permits unrestricted use, distribution and reproduction in any medium, provided the original work is properly cited.

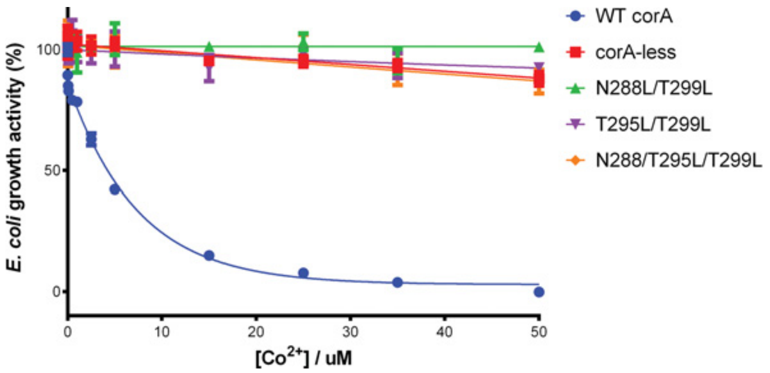

Figure S3 Co^{2+} transport assay of leucine double and triple mutants

The growth activity of TmCorA mutants was monitored in the presence of various Co^{2+} concentrations. A reduction in growth activity upon Co^{2+} concentration increase is indicative of the Co^{2+} transport activity of the TmCorA variant. The wild-type TmCorA (WT corA) and the empty CorA-less pBAD vector (corA-less) were used as positive and negative controls respectively. The results are the means±S.D. of at least three independent experiments.

Received 20 November 2012/24 January 2013; accepted 21 February 2013
Published as BJ Immediate Publication 21 February 2013, doi:10.1042/BJ20121745

© 2013 The Author(s)
The author(s) has paid for this article to be freely available under the terms of the Creative Commons Attribution Licence (CC-BY) (http://creativecommons.org/licenses/by/3.0/) which permits unrestricted use, distribution and reproduction in any medium, provided the original work is properly cited.
